# Reconciling the Entomological Hazard and Disease Risk in the Lyme Disease System

**DOI:** 10.3390/ijerph15051048

**Published:** 2018-05-22

**Authors:** Max McClure, Maria Diuk-Wasser

**Affiliations:** 1 Vagelos College of Physicians & Surgeons, Columbia University, New York, NY 10032, USA; mam2477@cumc.columbia.edu; 2 Department of Ecology, Evolution, and Environmental Biology, Columbia University, New York, NY 10027, USA

**Keywords:** Lyme disease, landscape, entomological risk, habitat fragmentation, land use, dilution effect, tick, biodiversity, *Ixodes scapularis*, *Borrelia burgdorferi*

## Abstract

Lyme disease (LD) is a commonly cited model for the link between habitat loss and/or fragmentation and disease emergence, based in part on studies showing that forest patch size is negatively related to LD entomological risk. An equivalent relationship has not, however, been shown between patch size and LD incidence (LDI). Because entomological risk is measured at the patch scale, while LDI is generally assessed in relation to aggregate landscape statistics such as forest cover, we posit that the contribution of individual patches to human LD risk has not yet been directly evaluated. We design a model that directly links theoretical entomological risk at the patch scale to larger-scale epidemiological data. We evaluate its predictions for relative LD risk in artificial landscapes with varying composition and configuration, and test its ability to predict countywide LDI in a 12-county region of New York. On simulated landscapes, we find that the model predicts a unimodal relationship between LD incidence and forest cover, mean patch size, and mean minimum distance (a measure of isolation), and a protective effect for percolation probability (a measure of connectivity). In New York, risk indices generated by this model are significantly related to countywide LDI. The results suggest that the lack of concordance between entomological risk and LDI may be partially resolved by this style of model.

## 1. Introduction

Lyme disease (LD), a tick-borne illness caused by the spirochete *Borrelia burgdorferi* s.s. and primarily transmitted in North America by the black-legged tick (*Ixodes scapularis*), is the most commonly reported vector-borne disease in the United States [1]. Since the 1960s or 1970s, the pathogen has expanded from its historic foci in the Northeast and Upper Midwest United States and has now been reported from 2203 American counties (over the period 2000–2016) and 109 Canadian municipalities (as of 2015) [1,2,3,4]. Following this spread, LD has become a model system for a number of hypotheses central to the field of disease ecology, including a proposed relationship between habitat loss and/or fragmentation and the risk of zoonosis emergence and human exposure [5].

At least two studies have found a correlation between forest patch size and entomological risk, with the latter measured as the density of infected *I. scapularis* nymphs (DIN). *I. scapularis* nymphs have been shown to play a critical role in the transmission of *B. burgdorferi* to humans, and DIN is widely used as an index of LD risk [6,7]. Under the assumption that tick density is directly related to the likelihood of a tick encounter, DIN approximates the probability of a human encountering a tick infected with *B. burgdorferi*. (On the pathogen invasion front, there is evidence that total nymph density may provide a better estimate of this infection risk [8].) Allan et al. found that DIN in forest patches in Dutchess County, New York, decreased with increasing forest patch area [9]. Brownstein et al. found a similar negative relationship between mean forest patch area and DIN in rural Connecticut towns [10]. Other studies have failed to find any correlation between patch size and DIN [11], which LoGiudice et al. attributed to an increased likelihood of tick population extinction in very small patches [12].

In addition to uncertainties in the relationship between patch size and DIN, the link between these hypothesized patch-level effects and larger-scale human disease incidence is equivocal. While the term “Lyme disease risk” is sometimes used to refer solely to entomological risk in the literature (e.g., [13,14]), entomological “risk” constitutes only the hazard, i.e., the source of harm to humans [15]. The relationship between this hazard and LD incidence is strongly mediated by human exposure to tick populations (both through incidental movement patterns and through deliberate preventive measures [16,17]), variations in human vulnerability to infection and disease once exposed, and reporting biases [18,19].

The relationship between DIN and LDI has been shown to vary in different states [20], and even direct measurements of DIN are not always correlated with human LDI in neighboring regions [21]. These intervening variables may partially explain why Brownstein et al. did not find that the large or highly connected forest patches associated with lower DIN in their study were protective against human LD. Instead, using traditional fragmentation metrics in Connecticut landscapes, the study found that mean patch isolation (defined by mean minimum distance between patch edges) was associated with exponentially decreasing LDI, while mean patch area was associated with exponentially increasing LDI [10]. 

A fundamental challenge in linking DIN with LDI is that DIN is necessarily measured at the patch scale while LDI is generally assessed as an aggregate statistic linked to a political unit, i.e., at the landscape scale. This disconnect in the representations of geographical space makes it difficult to assess how the processes driving the hazard and those driving human exposure are related. For example, the hazard may be greater in smaller patches, but aggregate LD incidence values may instead detect reduced LD risk because the population as a whole may be less likely to encounter these small patches [22]. An interplay between these opposing forces is suggested by an analysis of 12 Maryland counties that found a negative quadratic relationship between percentage forest cover and LDI, though forest cover is an imperfect proxy for patch area [23]. Furthermore, the relationship between hazard and risk may change at different levels of forest cover and patch connectivity.

We considered several versions of a simple model that incorporates both theoretical patch-level entomological risk and theoretical exposure. Using model output, we investigated LD risk indices for artificial landscapes at different forest cover percentages and levels of fragmentation, examining the relationship of a risk index dependent on a patch-level metric—patch size—to aggregate landscape statistics. We then tested our models’ ability to predict countywide LD incidence in a 12-county LD-endemic region of New York.

## 2. Materials and Methods

### 2.1. LD Risk Model

To generate a LD risk index, we first assumed that all LD cases were the result of peridomestic exposure—a widely made assumption in landscape analyses of LD risk based on the finding that approximately 2/3 of suburban and rural LD exposures in LD-endemic regions occur near the home [24]. For a population unit of interest (e.g., census tract, county), we considered the “peridomestic” range of that population unit, which could either be delineated by the borders of the unit or by a buffer around those borders.

We then considered all forest patches that fall within this range, assigning each patch a LD risk index proportional to the product of its associated entomological risk (hazard) and the probability of human exposure to said hazard (detailed below). This calculation assumes a multiplicative relationship between entomological risk and exposure, treating the two probabilities as independent of each other.

LD risk indices for all patches were then summed, yielding a total risk index for the population unit of interest. In order to translate this index to a larger population scale (as, for instance, when LD case data is available only at the county level, while population and landscape data are available at finer resolutions), LD risk indices for all population units of interest were then summed. The corresponding equation is:

(1) $$Risk=\overset{I}{\underset{i=1}{\sum }}(P_{i}\overset{J_{i}}{\underset{j=1}{\sum }}Exp_{j}Ent_{j})$$

where *i* = an individual population unit; *I* = total number of population units; *P_i_* = the population of population unit *i*; *j* = an individual patch within the range of population unit *i*; *J_i_* = total number of patches within range of population unit *i*; *Exp_j_* = the human risk of exposure to patch *j*; *Ent_j_* = the entomological risk associated with patch *j.*

### 2.2. Entomological Risk and Human Exposure Functions

The general framework described above allows comparison of several alternate models for exposure and/or entomological risk:

- We considered *Exp_j_* as either proportional to the perimeter of patch *j* that falls within range of *i* (proportional to the probability of entering patch *j*, assuming humans in population unit *i* move by random walk), proportional to the area of patch *j* that falls within the range of *i* (proportional to the relative amount of time spent in patch *j*), or as a constant.
- We considered *Ent_j_* as either a negative exponential function of the area of patch *j* (as hypothesized in Allan et al., 2003 [9]), a linear function of the area of patch *j*, or as a constant.

### 2.3. Simulated Landscapes

**Figure 1:**
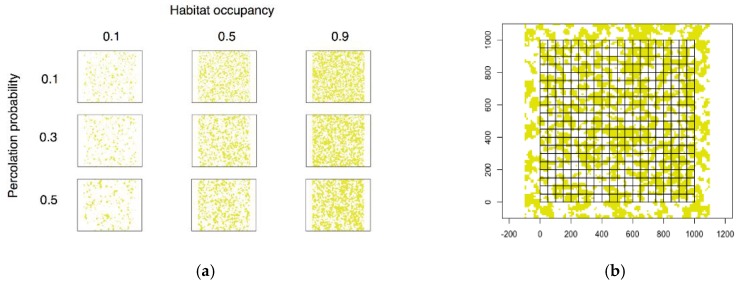
Simulated landscapes. (**a**) Modified Random Clusters landscapes with varying habitat (forest) occupancy and percolation probability. Forest cells are yellow, non-forest are white; (**b**) Simulated landscape with overlaid quadrats.

We used simulated landscapes to examine how hypothesized relationships between DIN and human exposure at the patch level translate to metrics measured at the landscape scale (e.g., LDI), in landscapes of varying composition and configuration. Using the Modified Random Clusters algorithm [25], we generated 120 × 120-cell raster artificial landscapes consisting of “habitat” (forest) and “non-habitat” land cover types, varying habitat occupancy *A* (defining landscape composition) and percolation probability *p* (defining landscape configuration) (Figure 1a).

In percolation theory, the value *p* represents the probability that a given site will be “occupied.” In the Modified Random Clusters algorithm, each cell of the generated artificial landscape is initially “marked” as potential habitat with this probability *p*. Contiguous clusters of marked cells are then identified and assigned habitat or non-habitat status so that the landscape-level habitat percentage equals *A*. Landscapes with lower *p* tend to feature many small habitat patches, while those with higher *p* tend to have fewer large patches. As a consequence, *p* is sometimes used as a measure of connectivity [26].

**Figure 2:**
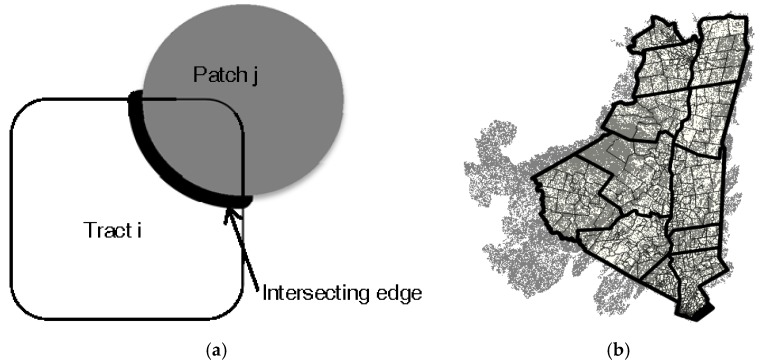
Model schematic and data. (**a**) Cartoon of model. The square represents the peridomestic region being considered. The gray circle represents an intersecting forest patch. When calculating entomological risk, the entire gray region is considered, as the patch’s entomological risk is a product of total area rather than area falling within the peridomestic region. When calculating exposure risk, only the portion of perimeter falling within the peridomestic region is considered (illustrated by bolded portion of perimeter); (**b**) Study landscape, including perimeter of 12-county region, 1.6 km buffer (black outer border in image), and intersecting forest patches (gray raster cells) derived from the National Land Cover Database 2011.

On each landscape, we overlaid a 20 × 20-quadrat grid of 5 × 5-cell quadrats to simulate population areas (Figure 1b). A 100-cell buffer between the borders of the grid and the borders of the landscape was left to avoid edge effects. We calculated the risk index of each quadrat according to the following version of our patch-risk model, which defines exposure (*Exp_j_*) as proportional to intersecting patch perimeter and entomological risk (*Ent_j_*) as a negative exponential function of patch area:

(2) $$\overset{J_{i}}{\underset{j=1}{\sum }}B_{x_{j}}e^{-A_{j}}$$

where $B_{x_{j}}$ = length of patch *j* perimeter intersecting tract *I*; $A_{j}$ = total area of patch *j* (Figure 2a). A 1-cell buffer was used when identifying the forest patches intersecting each quadrat. Assuming the non-habitat area was proportional to human occupancy, we defined the population of each quadrat *P_i_* as the number of non-habitat (i.e., human-occupied) cells it contained. Landscape-level LD risks for each simulation were calculated by summing risk indices for all quadrats and then dividing by total population.

### 2.4. Landscape Analysis

Landcover data was obtained from the National Land Cover Database 2011 (from the Multi-Resolution Land Characteristics Consortium) in the form of a 16-class landcover-classified 30 m-resolution raster layer [27]. Forest patches, defined as 8-neighbor contiguous clusters of deciduous forest or mixed forest cells (land classes 41 and 43) with area greater than >1 ha (to approximate the restrictions used in Allan, et al., 2003 [9]), were identified using QGIS ver. 2.18.14 (Open Space Geospatial Foundation, Beaverton, OR, USA) and R ver. 3.3.2 (R Foundation, Vienna, Austria).

The extent of the study region was defined as the perimeter of the 12 counties identified above, with an additional 2.4 km buffer (based on the buffer definition used in a prior study of LD risk in the wildland-urban interface [28]). All forest patches that intersected this extent were included in subsequent analysis (Figure 2b). Further landscape analysis was conducted in R using the rgdal, rgeos, spdep, raster, and SDMTools libraries.

### 2.5. Risk Index Calculation

For risk index calculation, we defined population units *P_i_* as U.S. census tracts, delineated by shapefiles obtained from the New York State Civil Boundaries dataset and using population data from the 2010 U.S. census [29]. All forest patches that intersected a 2.4 km buffer surrounding each census tract contributed to that tract’s risk indices (again based on Larsen, et al., 2014 [28]).

We calculated risk indices for each tract using all possible combinations of the three formulae for human exposure risk and three formulae for entomological risk described above. County-level risk indices were then generated by summing risk indices across all census tracts within each county and dividing by county population. Formulae were as follows:

- exposure constant, entomological risk as a negative exponential function of patch area:
  (3) $$\overset{I}{\underset{i=1}{\sum }}(P_{i}\overset{J_{i}}{\underset{j=1}{\sum }}1\times e^{-A_{j}})$$
- exposure directly related to intersecting patch perimeter, entomological risk as a negative exponential function of patch area:
  (4) $$\overset{I}{\underset{i=1}{\sum }}(P_{i}\overset{J_{i}}{\underset{j=1}{\sum }}B_{x_{j}}e^{-A_{j}})$$
- exposure directly related to intersecting patch area, entomological risk as a negative exponential function of patch area:
  (5) $$\overset{I}{\underset{i=1}{\sum }}(P_{i}\overset{J_{i}}{\underset{j=1}{\sum }}A_{x_{j}}e^{-A_{j}})$$
- exposure directly related to intersecting patch perimeter, entomological risk constant:
  (6) $$\overset{I}{\underset{i=1}{\sum }}(P_{i}\overset{J_{i}}{\underset{j=1}{\sum }}B_{x_{j}}\times 1)$$
- exposure directly related to intersecting patch area, entomological risk constant:
  (7) $$\overset{I}{\underset{i=1}{\sum }}(P_{i}\overset{J_{i}}{\underset{j=1}{\sum }}A_{x_{j}}\times 1)$$
- exposure constant, entomological risk directly related to area:
  (8) $$\overset{I}{\underset{i=1}{\sum }}(P_{i}\overset{J_{i}}{\underset{j=1}{\sum }}1\times A_{j})$$
- exposure directly related to intersecting patch perimeter, entomological risk directly related to area:
  (9) $$\overset{I}{\underset{i=1}{\sum }}(P_{i}\overset{J_{i}}{\underset{j=1}{\sum }}B_{x_{j}}A_{j})$$
- exposure directly related to intersecting patch area, entomological risk directly related to area:
  (10) $$\overset{I}{\underset{i=1}{\sum }}(P_{i}\overset{J_{i}}{\underset{j=1}{\sum }}A_{x_{j}}A_{j})$$

where *I* = number of tracts; *J_i_* = number of forest patches that intersect tract *i*; $P_{i}$ = population of tract *i*; $B_{x_{j}}$ = for patch *j*, length of perimeter intersecting tract *i*; $A_{x_{j}}$ = for patch *j*, area intersecting tract *i*; $B_{j}$ = total perimeter of patch *j*; and $A_{j}$ = total area of patch *j*.

Aggregate risk indices were calculated for these same formula combinations, using total or mean forest area and perimeter rather than (summed) patch-level data. Entomological risk was modeled as either a negative exponential or linear function of total forest patch area *A* or mean patch area *E*[*A*]. Exposure was modeled as a constant or as proportional to total or mean patch area or perimeter (*B* or *E*[*B*]). We additionally considered percent forest cover per county and total number of forest patches per county.

Although we framed *Ent_j_* and *Exp_j_* as separate, our model is incapable of distinguishing between entomological risk and exposure when applied to real landscapes. In other words, although we may conceptualize Equation (3) as representing entomological risk in the form of a negative exponential function of patch area and exposure as a constant, it equally well represents the inverse.

### 2.6. County LDI

LD case counts were obtained from Centers for Disease Control and Prevention (CDC, Atlanta, GA, USA) passive surveillance data on countywide LD case totals over the period 2000–2015 [1]. The reporting case definition of LD changed twice over this period. Notable changes are as follows: beginning in 2008, reported cases were expanded to include patients with erythema migrans in the setting of “known exposure” to potential tick habitat, as well as “probable” cases defined by physician-diagnosed LD with laboratory confirmation; and, beginning in 2011, the definition of “laboratory confirmation” was expanded to include positive cerebrospinal fluid antibody tests [30]. All changes were made at a national scale and did not vary between counties. County-level variation in physician diagnosis or reporting rates are possible, but were not investigated.

LDI over the study period was calculated by summing LD cases across all years and dividing by county population derived from the 2010 US Census and matched to county shapefiles from the New York State Civil Boundaries dataset [29]. To ensure only counties in which LD was endemic were considered, analysis was restricted to 12 contiguous counties that had reported >1 case of LD in every year surveilled (Albany, Columbia, Dutchess, Greene, Orange, Putnam, Rensselaer, Rockland, Schenectady, Sullivan, Ulster, and Westchester). 

### 2.7. Model Evaluation

The resulting set of LDIs was log-transformed to satisfy normality and stabilize variance, following the procedure of Waller and Gotway [31]. Spatial structure of LDI was analyzed for autocorrelation using global Moran’s I and correlograms [32].

We evaluated spatial dependence of the transformed LDI on county-level LD risk indices using simultaneous autoregressive models—a method that accounts for spatial dependence by regressing the error terms for each area on the error terms for neighboring areas. We assumed normal error structure. Binary spatial weights were defined by contiguity and row-standardized. County LDI was weighted proportionally to the inverse of county population. Models were estimated by maximum likelihood using the spdep package in R and residuals were analyzed for autocorrelation using local Moran’s I.

## 3. Results

### 3.1. Simulated Landscapes

In simulated landscapes, forest patch area distributions were consistently right-skewed at all but the highest levels of habitat occupancy. Mean minimum distance between patch edges decreased with habitat occupancy and increased with percolation probability. Mean patch size increased with habitat occupancy and exhibited no clear relationship to percolation probability. The number of patches decreased with percolation probability and exhibited a unimodal relationship with habitat occupancy (Figure S1).

**Figure 3:**
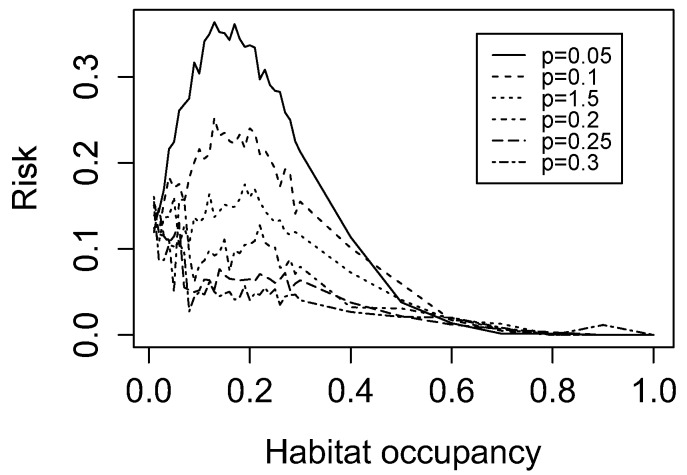
Lyme disease (LD) risk of simulated landscapes. Predicted LD risk as a function of habitat occupancy (forest cover). Each curve is evaluated for a landscape with a different percolation probability *p*.

At low percolation probabilities, a strong relationship was seen between habitat occupancy and landscape-level LD risk: predicted LD risk increased from a value of 0 at 0% forest cover (not shown) to a peak near 20% cover, and then decreased consistently as cover increased. These trends were attenuated by increasing percolation probability (Figure 3). The same patterns were observed when one-cell patches were excluded from the analysis to simulate tick or pathogen extinction in very small forest patches, although LD risk scores were universally lower (Figure S2).

**Figure 4:**
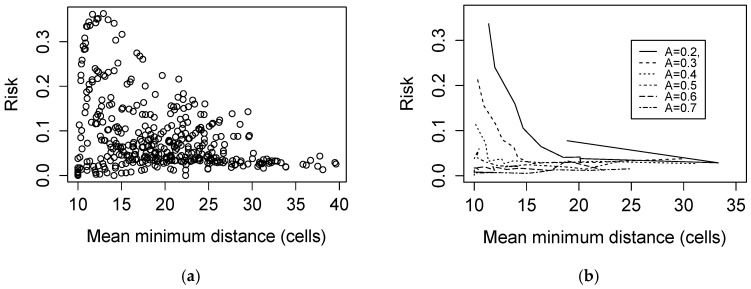
Predicted Lyme disease risk as a function of mean minimum distance between patch edges, (**a**) across all simulated landscapes and (**b**) stratified by habitat occupancy *A*. Each curve connects landscape scores evaluated at sequential values of percolation probability *p* (0.05–0.5)*.*

Comparing the model-predicted LD risk to traditional fragmentation metrics as used in Brownstein et al. [10], predicted LD risk exhibited a right-skewed unimodal relationship with mean minimum distance between patch edges. When analysis was stratified by level of habitat occupancy, LD risk took on a negative relationship to mean minimum distance for each level of habitat occupancy (Figure 4).

**Figure 5:**
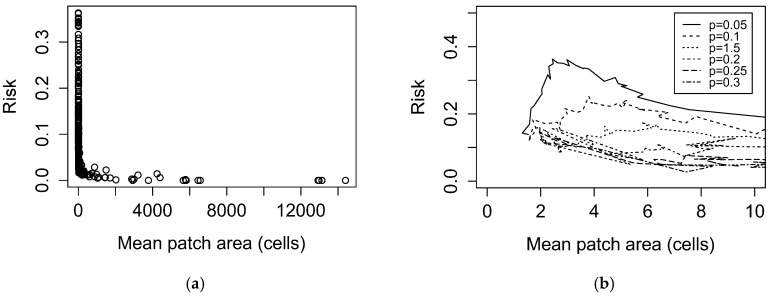
Predicted Lyme disease risk as a function of mean patch area. (**a**) LDI as a function of patch area across all simulated landscapes; (**b**) LDI as a function of area stratified by percolation probability *p*, with axes rescaled to better show maxima. Each curve connects landscape scores evaluated at sequential values of habitat occupancy *A* (0.1–0.9).

Predicted LD risk exhibited a heavily right-skewed unimodal relationship with mean patch area. This relationship was attenuated by increasing percolation probability (Figure 5). Predicted LD risk increased with the number of forest patches of all sizes (Figure S3).

### 3.2. Landscape Characterization

As with the simulated landscapes above, distribution of forest patch areas in the 12 NY counties was weighted heavily towards smaller patches (area < 6 ha) (Figure S4). For countywide aggregate statistics, see Table 1.

Among forest patches that intersected a given census tract, intersecting forest perimeter $\sum_{j=1}^{J}B_{x_{j}}$ was highly correlated (ρ = 1.00) with intersecting forest area $\sum_{j=1}^{J}A_{x_{j}}$. $\sum_{j=1}^{J}B_{x_{j}}$ exhibited moderate correlation with the total perimeter of intersecting patches $\sum_{j=1}^{J}B_{j}$ (ρ = 0.504). $\sum_{j=1}^{J}A_{x_{j}}$ was similarly moderately correlated with the total area of intersecting patches $\sum_{j=1}^{J}A_{j}$ (ρ = 0.527).

### 3.3. Spatial Structure of LDI

Countywide LDI showed significant global spatial autocorrelation (Moran’s I = 1.648, *p* = 0.050). Areal correlogram showed no trend in the relationship between autocorrelation and lag (Figure S5). Over the study period, highest total LD case counts were seen in Dutchess County, while highest mean LDI was seen in Columbia County (Table 1).

### 3.4. Model Evaluation

The best-performing class of LD risk indices were those generated by tract-level models that treated entomological risk as a negative exponential function of forest patch area (Table 2). All models in this class were significantly predictive of countywide LDI as defined above (*p* < 0.01), and performed better than all other classes of tested models by log-likelihood. Without taking spatial weights into account, all models in this class featured *R*^2^ of 0.67–0.68 (Table 2).

Tract-level models that defined entomological risk as a linear function of forest patch area or as a constant did not significantly explain LDI distribution.The definition of human exposure had no consistent effect on model performance. 

Seven of the risk indices generated using countywide aggregate data were significantly explanatory (*p* < 0.05) (Table 3), but resulted in lower log-likelihoods than models in the negative-exponential tract-level class.

Regardless of their explanatory power, all tested variables resulted in non-significant spatial autocorrelation parameter λ (*p* > 0.05), indicating that the residuals exhibited no significant global spatial autocorrelation. By local Moran’s I, all significantly explanatory models showed a single cluster of residuals centered on Greene county (pseudo-*p* ≤ 0.01).

## 4. Discussion

The primary model tested here assumes both that there is a negative exponential relationship between forest patch area and entomological risk, and that entomological risk is related to local human LD incidence. Using simulated landscapes, we show how this relationship at the patch scale can be translated into landscape metrics, resulting in the nonlinear relationship between forest cover and LDI shown empirically in previous studies [23]. We propose this paradigm as an initial step to linking patch and landscape level processes, acknowledging that it is a simplification of the real spatial epidemiology of LD that ignores the role of non-forest land cover, movement and population sizes of deer and other hosts, and human behavioral variation and control measures [5].

The mechanism underlying this hypothesized pattern of increased risk with decreased forest area is often identified as a component of the “dilution effect” hypothesis. Originally formulated by Ostfeld, Keesing and others based on studies conducted in upstate New York [33,34,35], the hypothesis holds that increased host diversity will be generally protective against infectious disease emergence and spillover of zoonotic pathogens by virtue of decreased contact rates between competent hosts. Regions with greater forested area are believed to house such increased diversity, as predicted by the species-area curve [36]. The accuracy and generalizability of both the dilution effect and its proposed mechanism have since been vigorously contested [22,37,38,39] and defended [40]. Alternate camps either question the effect’s existence or adhere to the hypothesis that degraded landscapes confer greater zoonosis risk because they support larger populations of generalist, pathogen-competent species, independent of biodiversity [22]. The potential decoupling of these various effects is supported by a recent study finding greater diversity of reservoir host species, similar white-footed mouse (i.e., competent host) populations, and reduced *B. burgdorferi* exposure in mice in mixed forest-suburban habitats, as opposed to large, intact forested stands [41].

Using simulated landscapes in which entomological risk is a negative exponential function of forest patch total area and human exposure risk is proportional to intersecting forest patch perimeter, we find that predicted LDI reaches a local maximum at ~20% habitat occupancy. Our result is consistent with one existing hypothesis of landscape fragmentation and LD risk, which holds that risk is negligible in highly urbanized areas, rises in residential areas and fragmented landscapes, and decreases again in large, intact forests [5]. This theoretical unimodal relationship has been corroborated in one study of several counties in Maryland, where the authors found a negative quadratic relationship between forest cover and LD risk [23]. The peak in this study was observed at 53.5% cover, which may reflect variation between the landscapes studied (e.g., fragmentation metrics not accounted for in their study), or more fundamental differences in real-world entomological risk or human exposure. This posited increase in LD risk at low or intermediate levels of forest cover has been cited as evidence against a naïve application of the proposed fragmentation-LD relationship [22,37]. Our analysis shows that a negative patch area-entomological risk relationship at the forest patch scale results in a unimodal relationship between LDI and forest cover at the landscape scale. This nonlinear, non-monotonic association is not always captured by aggregate landscape statistics.

We additionally find that this unimodal pattern is only present below a percolation probability threshold (approximately *p* = 0.25 in our artificial landscapes), granting increased forest connectivity a potential protective effect. The shape of the curve is unaffected by removal of very small patches, implying that increased extinction rates of tick populations below a forest patch area threshold (as hypothesized by LoGiudice et al.) would not result in a qualitatively different relationship between forest patch distribution and LD risk at the landscape scale [12].

Our findings on simulated landscapes are variably consistent with results generated by traditional fragmentation metrics in Brownstein, et al. [10]. We do not find the increasing exponential relationship between mean patch area and LD risk the authors describe in Connecticut forest patches, although there is a small range of patch areas (approximately 1–3 cells) for which predicted LD risk does increase with patch area in low-percolation probability landscapes. As before, this inconsistency may reflect inaccuracies in our model of either entomological risk or human exposure.

Our results are, however, consistent with the same study’s observation that mean minimum distance between patches is related to LD risk by a negative exponential. Our simulated results generate a similar relationship either when results are stratified by habitat occupancy, or when only considering landscapes above a mean minimum distance threshold (approximately >10 cells). In other words, our LD risk model may be consistent with the relationship Brownstein et al., observe if their Connecticut study area did not incorporate a wide range of forest cover percentages between landscapes, or if these landscapes only included forest patches separated by a mean minimum distance above the aforementioned threshold. Note that all results derived from our artificial landscapes are to some extent dependent on the specific landscape-generating algorithm used here, and should be reexamined on simulated landscapes produced with other methods.

Applying our model to real-world LDI in New York, we find that a negative exponential forest patch area-entomological risk relationship at the census tract level generates risk indices that are significantly predictive of countywide LDI spatial distribution. These results are generated at a coarse scale with a small sample size and explain roughly 2/3 of observed LDI variation, but broadly support the idea that a negative relationship between forest patch size and entomological risk plays a role in determining human case distribution. They do not necessarily constitute evidence that the patch area-DIN relationship is best represented by the negative exponential function tested here. For example, the significant relationship between a simple count of forest patches and LDI at the county level may be consistent with either our model (as suggested by the relationship between number of patches and predicted LDI in Figure S3), or with a direct effect of number of patches of a given size.

As noted in Section 2.5, the result is also equally consistent with an equivalent relationship between patch area and exposure, rather than entomological risk. We do not see the negative quadratic relationship between forest cover and LDI reported by Jackson et al. [23] and suggested by our results on artificial landscapes, nor a relationship between mean patch area and LDI as reported by Brownstein et al.. This difference may reflect variation in the scale of analysis, in the range of composition exhibited by the respective study landscapes, or in the influence of matrix or habitat quality.

Our findings are robust to assumptions about exposure risk at the scale of our study. This result is likely predicated in part on the high degree of correlation between intersecting perimeter and intersecting area in this landscape. These results do not replace or preclude more detailed considerations of human behavior, including evaluation of leisure time, employment, and travel history in order to more precisely account for variation among individual humans, patch-level characteristics that may drive human visit frequency, and exposure to LD outside the peridomestic region. If, for instance, humans are much more or much less likely to visit patches of a certain size than their area or perimeter would imply, our assumption of random movement in this study would lead to large errors in risk estimation. Similarly, our assumption that entomological risk and exposure were independent of each other (and therefore related by multiplication) does not allow for the possibility that tick populations affect human visit rates and vice versa. The explicit inclusion of human movement data in future models, as well as risk maps that integrate ecological risk factors and social and behavioral patterns, will allow for a more precise accounting of these interacting mechanisms [42].

While our framing represents a reasonable encapsulation of the association between patch size and DIN proposed as supporting the dilution effect hypothesis, we do not provide evidence of specific mechanisms (i.e., host composition changes) driving this relationship. Also, because we do not directly consider DIN data or census tract-level incidence, our analysis is unable to evaluate whether our models’ explanatory power derives from the proposed relationship between patch size and entomological risk. To field-validate the proposed mechanism underlying this approach, it will be necessary to directly link forest patch size to tick surveillance data, including DIN and, in emerging areas, density of nymphs, and model output to finer-scale (e.g., census tract-, town- or ZIPcode™-level) local LDI. Analysis of land use change time series and concurrent trends in LDI would also strengthen the hypothetical relationship. Future studies should evaluate whether the model will be similarly predictive in other landscapes, systematically assessing which forms of the model and which parameters provide the best performance. 

## 5. Conclusions

We find that a negative exponential relationship between forest patch size and entomological risk is consistent with human LD case distribution in southern New York state, and that the implications of this relationship are consistent with current qualitative models of the interaction between habitat fragmentation and LD. This analysis suggests that the apparent paradox in the LD literature, in which landscape features that increase entomological risk are occasionally found to decrease human LDI, may be resolved by directly accounting for the interaction of patch-level entomological risk with human exposure.

## Figures and Tables

**Table 1:** County-level forest and LD statistics. Forest patch statistics only include deciduous and mixed forest patches with areas greater than 1 ha.

County	Population 2010	Mean LDI 2000–2015	Forest Area (ha) 2011	Forest Perimeter (km) 2011	No. Patches 2011
Albany	304,204	7.40 × 10^−4^	65.1426	378.75	1374
Columbia	63,096	6.76 × 10^−3^	83.0822	407.078	1637
Dutchess	297,488	2.49 × 10^−3^	111.7104	516.086	1835
Greene	49,221	3.12 × 10^−3^	125.3981	410.046	747
Orange	372,813	1.06 × 10^−3^	118.2992	446.79	1853
Putnam	99,710	1.66 × 10^−3^	43.4138	157.504	349
Rensselaer	159,429	1.44 × 10^−3^	91.6401	455.258	1560
Rockland	311,687	5.30 × 10^−4^	21.4342	71.91	548
Schenectady	154,727	1.70 × 10^−4^	26.1887	142.528	548
Sullivan	77,547	4.62 × 10^−4^	204.5404	604.91	539
Ulster	182,493	1.73 × 10^−3^	228.011	598.846	1241
Westchester	949,113	3.06 × 10^−4^	47.8913	267.724	1150

**Table 2:** Explanatory power of tract-level landscape variables.

Formula		*Exp_j_*	*Ent_j_* ~ Area	Coefficient	SE	*p*	*λ*	*p_λ_*	*L*	*R* ^2^
$\overset{I}{\underset{i=1}{\sum }}(P_{i}\overset{J_{i}}{\underset{j=1}{\sum }}e^{-A_{j}})$	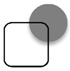	Const.	Neg. Exp.	5.47 × 10^−2^	1.01 × 10^−2^	5.70 × 10^−8^ ***	−2.43 × 10^−2^	9.39 × 10^−1^	−10.0	0.68
$\overset{I}{\underset{i=1}{\sum }}(P_{i}\overset{J_{i}}{\underset{j=1}{\sum }}B_{x_{j}}e^{-A_{j}})$	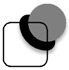	Perim.	Neg. Exp.	2.55 × 10^−2^	5.01 × 10^−3^	3.66 × 10^−7^ ***	−7.85 × 10^−3^	9.80 × 10^−1^	−10.5	0.67
$\overset{I}{\underset{i=1}{\sum }}(P_{i}\overset{J_{i}}{\underset{j=1}{\sum }}A_{x_{j}}e^{-A_{j}})$	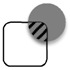	Area	Neg. Exp.	8.43 × 10^−1^	1.67 × 10^−1^	4.76 × 10^−7^ ***	−9.00 × 10^−4^	9.98 × 10^−1^	−10.5	0.67
$\overset{I}{\underset{i=1}{\sum }}(P_{i}\overset{J_{i}}{\underset{j=1}{\sum }}B_{x_{j}})$	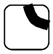	Perim.	Const.	1.95 × 10^−2^	1.31 × 10^−2^	1.38 × 10^−1^	3.76 × 10^−1^	5.56 × 10^−1^	−15.1	0.16
$\overset{I}{\underset{i=1}{\sum }}(P_{i}\overset{J_{i}}{\underset{j=1}{\sum }}A_{x_{j}})$	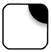	Area	Const.	5.83 × 10^−4^	3.94 × 10^−4^	1.39 × 10^−1^	3.77 × 10^−1^	5.75 × 10^−1^	−15.1	0.16
$\overset{I}{\underset{i=1}{\sum }}(P_{i}\overset{J_{i}}{\underset{j=1}{\sum }}A_{j})$	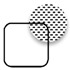	Const.	Lin.	1.53 × 10^−5^	2.96 × 10^−4^	9.59 × 10^−1^	6.45 × 10^−1^	1.32 × 10^−1^	−15.7	0.016
$\overset{I}{\underset{i=1}{\sum }}(P_{i}\overset{J_{i}}{\underset{j=1}{\sum }}B_{x_{j}}A_{j})$	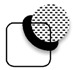	Perim.	Lin.	−1.56 × 10^−8^	1.82 × 10^−7^	9.32 × 10^−1^	6.59 × 10^−1^	1.24 × 10^−1^	−15.7	0.025
$\overset{I}{\underset{i=1}{\sum }}(P_{i}\overset{J_{i}}{\underset{j=1}{\sum }}A_{x_{j}}A_{j})$	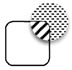	Area	Lin.	−5.21 × 10^−7^	6.07 × 10^−6^	9.32 × 10^−1^	6.59 × 10^−1^	1.24 × 10^−1^	−15.7	0.025
No. patches < 2 ha				1.35 × 10^−1^	2.62 × 10^−2^	2.46 × 10^−7^ ***	−1.36 × 10^−2^	9.66 × 10^−1^	−10.3	0.67

Cartoons of tract-level risk indices are depicted to the right of the corresponding formula, where square represents census tract, circle represents forest patch, gray areas are subject to a negative exponential function, stippled areas are subject to a linear function, and overlap is indicated by diagonal lines. *I* = number of tracts; *J_i_* = number of forest patches that intersect tract *i*; $P_{i}$ = population of tract *i*; $B_{X_{i}}$ = for patch *j*, length of perimeter intersecting tract *i*; $A_{X_{i}}$ = for patch *j*, area intersecting tract *i*; $B_{j}$ = total perimeter of patch *j*; = total area of patch *j*; *λ* = spatial autocorrelation parameter; *p_λ_* = *p* value associated with *λ*; *L* = log-likelihood. “***” indicates significance to *p* < 0.001.

**Table 3:** Explanatory power of county-level landscape risk indices.

Formula	Coefficient	SE	*p*	*λ*	*p_λ_*	*L*
$e^{-A}$	−2.92 × 10^0^	8.61 × 10^−1^	6.95 × 10^−4^ ***	−1.20 × 10^−1^	7.62 × 10^−1^	−13.8
$Be^{-A}$	−2.57 × 10^0^	3.54 × 10^0^	4.67 × 10^−1^	6.85 × 10^−1^	5.34 × 10^−2^	−15.5
$Ae^{-A}$	1.25 × 10^1^	2.98 × 10^0^	2.60 × 10^−5^ ***	−6.98 × 10^−1^	3.45 × 10^−1^	−15.3
$e^{-E\left[A\right]}$	−1.72 × 10^0^	1.26 × 10^0^	1.73 × 10^−1^	5.41 × 10^−1^	1.94 × 10^−1^	−14.9
$E\left[B\right]e^{-E\left[A\right]}$	−4.25 × 10^0^	5.96 × 10^0^	4.76 × 10^−1^	6.57 × 10^−1^	6.43 × 10^−2^	−15.5
$E\left[A\right]e^{-E\left[A\right]}$	7.26 × 10^0^	3.53 × 10^0^	3.98 × 10^−2^ *	6.50 × 10^−1^	6.26 × 10^−2^	−13.9
E[A]	1.03 × 10^−1^	3.4 × 10^−1^	7.64 × 10^−1^	6.27 × 10^−1^	1.14 × 10^−1^	−15.7
*A*	8.74 × 10^−4^	4.07 × 10^−4^	3.17 × 10^−2^ *	1.47 × 10^−1^	7.39 × 10^−1^	−14.8
% Forest cover	3.62 × 10^3^	1.57 × 10^3^	2.14 × 10^−2^ *	4.79 × 10^−1^	2.60 × 10^−1^	−13.7
(% Forest cover) ^2^	2.79 × 10^6^	1.47 × 10^6^	5.79 × 10^−1^	5.05 × 10^−1^	2.33 × 10^−1^	−14.3
*B*	3.89 × 10^−3^	1.04 × 10^−3^	1.96 × 10^−4^ ***	−2.94 × 10^−1^	4.40 × 10^−1^	−14.0
No. patches	1.75 × 10^−3^	3.40 × 10^−4^	2.93 × 10^−7^ ***	−7.70 × 10^−1^	9.47 × 10^−2^	−14.2

*A* = total forest area in county; *B* = total forest perimeter in county; *E*[x] indicates a mean; *λ* = spatial autocorrelation parameter; *p_λ_* = *p* value associated with *λ*; *L* = log-likelihood. “***” indicates significance to *p* < 0.001, “*” indicates significance to *p* < 0.05; ^2^: indicates that the percentage is raised to the second power (a quadratic function).

## Supplementary Materials

The following are available online at http://www.mdpi.com/1660-4601/15/5/1048/s1, Figure S1: Relations between Modified Random Clusters parameters and traditional fragmentation statistics in simulated landscapes, Figure S2: Predicted LD risk of simulated landscapes (excluding one-cell forest patches) as a function of habitat occupancy, Figure S3: Predicted LD risk as a function of number of forest patches, Figure S4: Frequency distribution of log-transformed deciduous and mixed forest patch areas, Figure S5: Lyme disease incidence correlogram.
