# Supplementary material for: Reconciling the Entomological Hazard and Disease Risk in the Lyme Disease System
Source: Int J Environ Res Public Health. 2018 May 22;15(5):1048. doi: 10.3390/ijerph15051048 (PMC5982087; doi:10.3390/ijerph15051048)
Supplement: Supplementary file 1 [file ijerph-15-01048-s001.pdf]

Supplementary Figures

Reconciling the Entomological Hazard and Disease Risk in the Lyme Disease System

Max McClure [1] and Maria Diuk-Wasser [2,*]

[1] Vagelos College of Physicians & Surgeons, Columbia University, New York City, NY 10032, USA; mam2477@cumc.columbia.edu

[2] Department of Ecology, Evolution, and Environmental Biology, Columbia University, New York City, NY 10027, USA

* Correspondence: mad2256@columbia.edu;

(a)

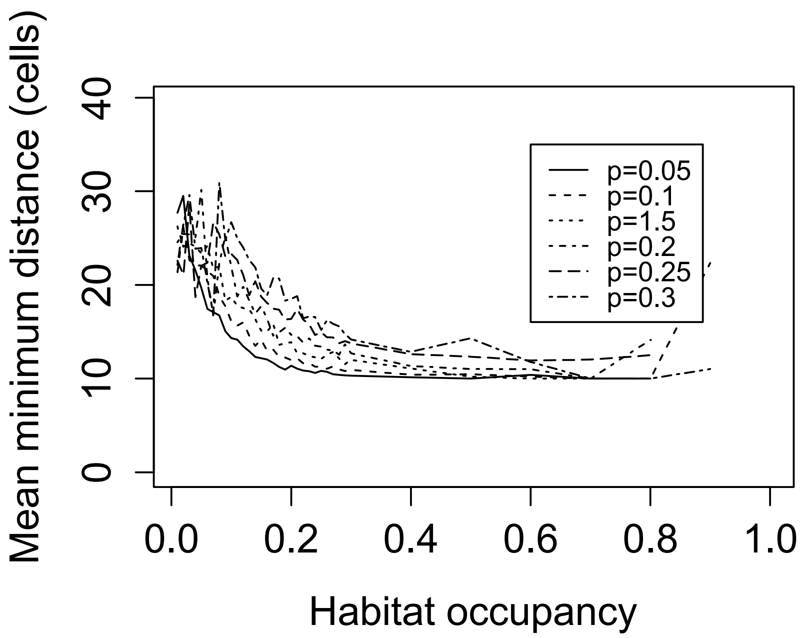

Figure S1. *Cont.*

(b)

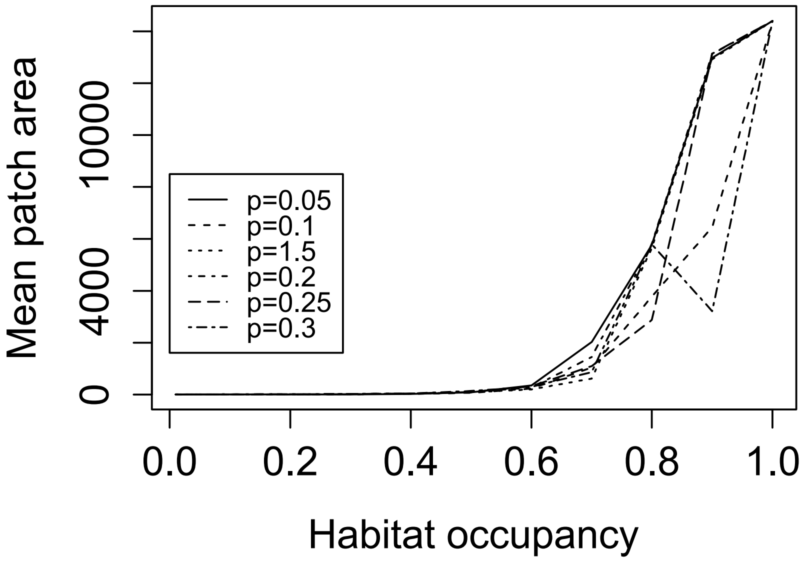

Mean patch area
0
4000
10000
0.0
0.2
0.4
0.6
0.8
1.0
Habitat occupancy
p=0.05
p=0.1
p=1.5
p=0.2
p=0.25
p=0.3

(c)

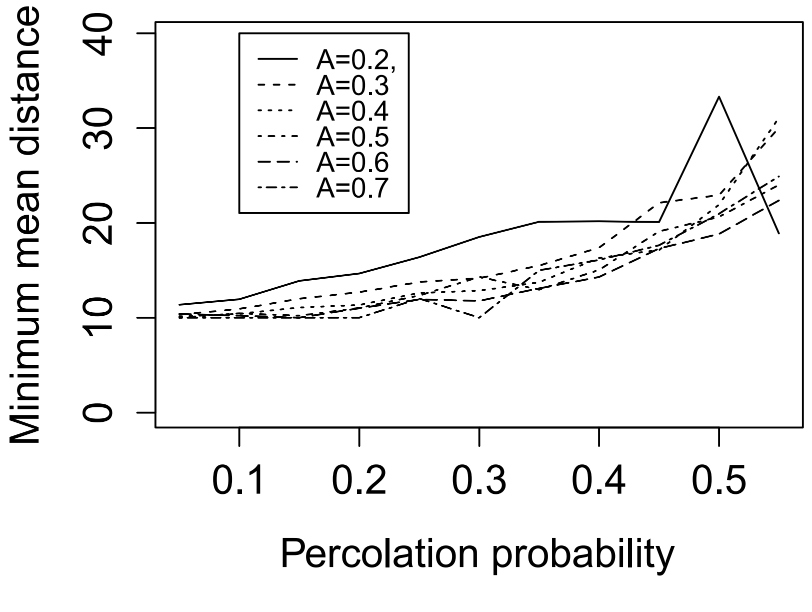

Minimum mean distance
0
10
20
30
40
0.1
0.2
0.3
0.4
0.5
Percolation probability
A=0.2,
A=0.3
A=0.4
A=0.5
A=0.6
A=0.7

Figure S1. *Cont.*

(d)

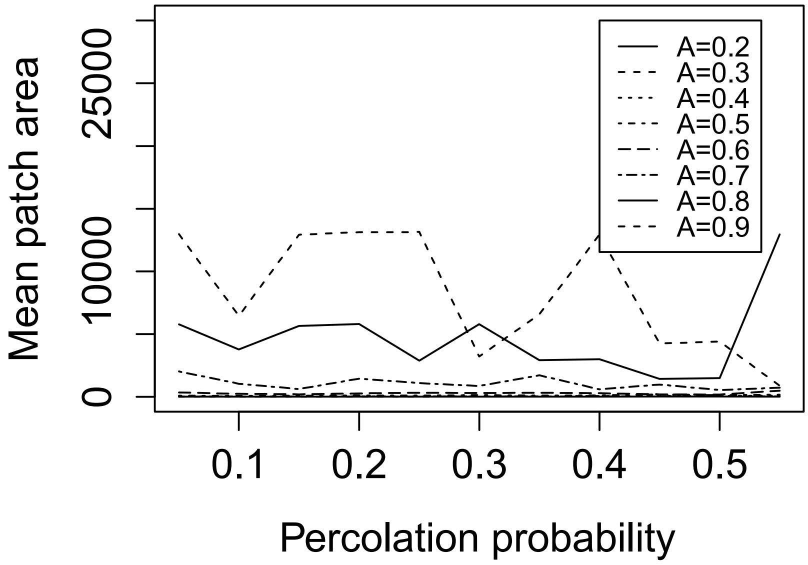

(e)

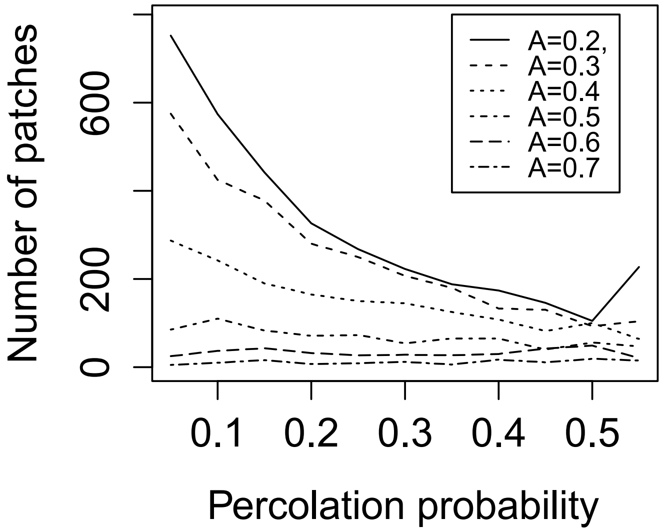

Figure S1. *Cont.*

(f)

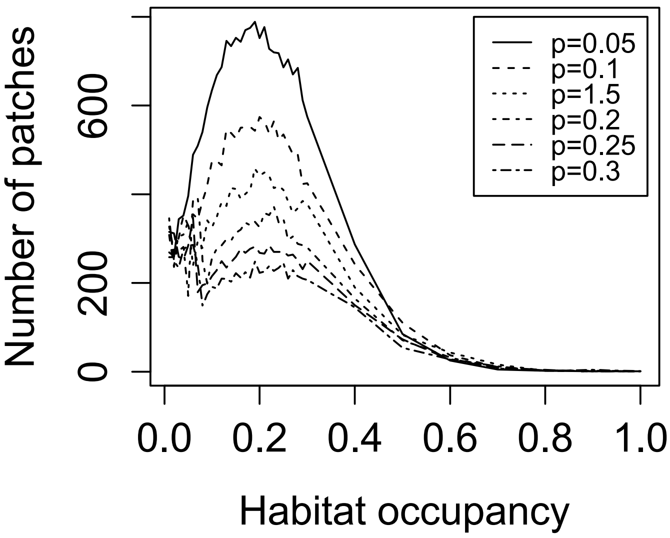

Figure S1. Relations between Modified Random Clusters parameters (habitat occupancy and percolation probability) and traditional fragmentation statistics in simulated landscapes. **(a)** Patch isolation as a function of habitat occupancy *A*. **(b)** Patch isolation as a function of percolation probability *p*. **(c)** Mean patch area as a function of *A*. **(d)** Mean patch area as a function of *p*. **(e)** Number of patches as a function of *p*. **(f)** Number of patches as a function of *A*.

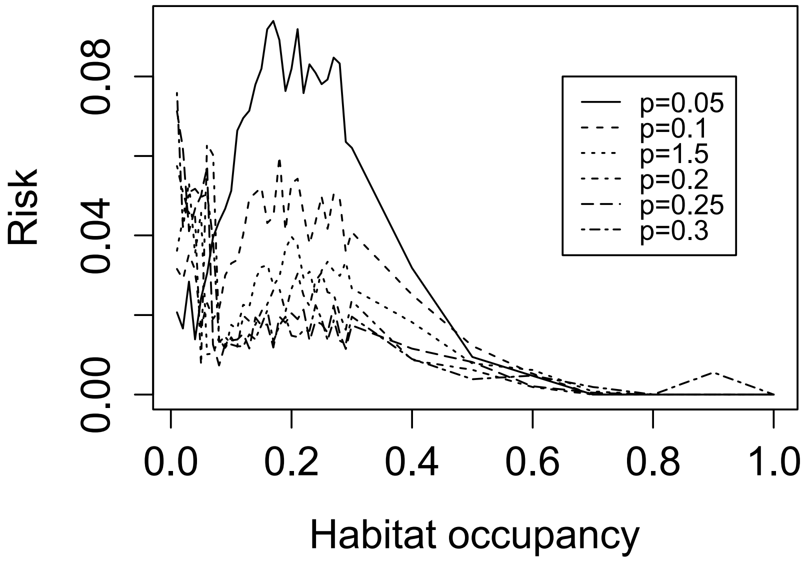

Figure S2. Predicted LD risk of simulated landscapes (excluding one-cell forest patches) as a function of habitat occupancy (forest cover). Each curve is evaluated for a landscape with a different percolation probability *p*.

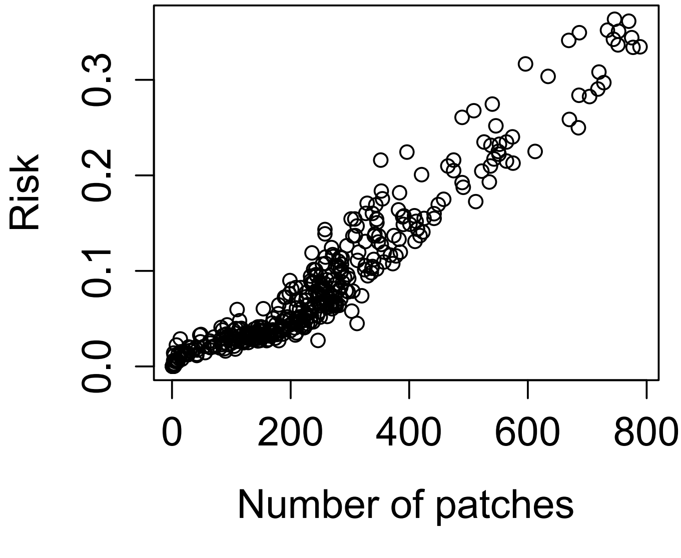

Figure S3. Predicted LD risk as a function of number of forest patches.

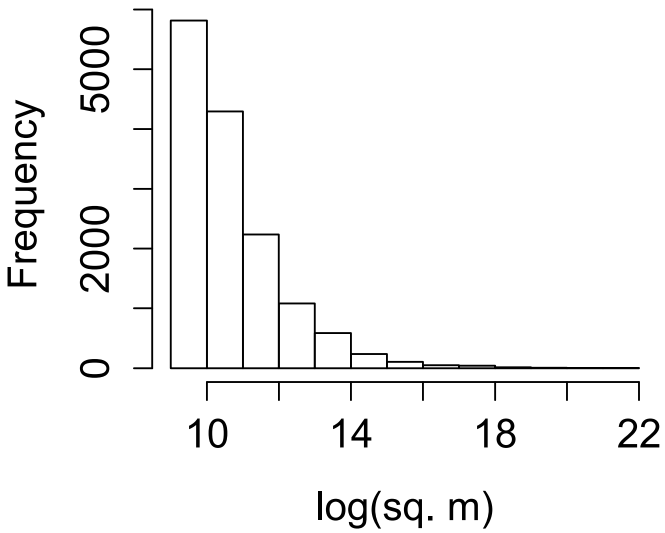

Figure S4. Frequency distribution of log-transformed deciduous and mixed forest patch areas.

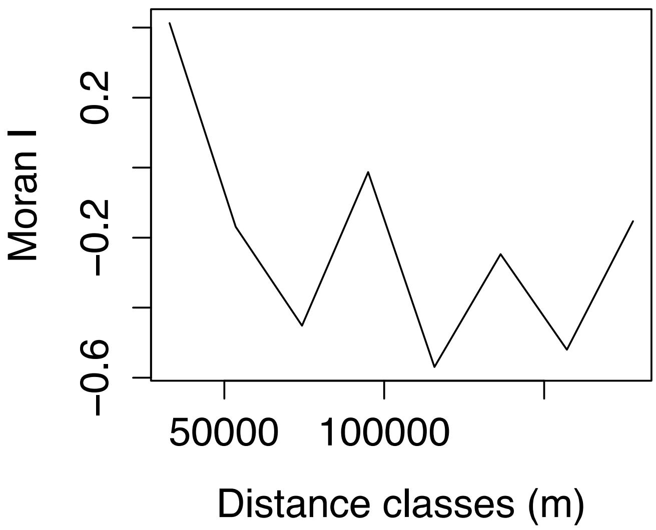

Figure S5. Lyme disease incidence (LDI) correlogram. Plot of Moran's I for LDI as a function of distance class within study region.
